# Pediatric Nasopharyngeal Carcinoma: Survival Outcomes and Late Toxicity Burden from a 20-Year Single-Center Experience

**DOI:** 10.3390/children13070896

**Published:** 2026-07-04

**Authors:** Mehtap Ertekin, Aytul Temuroglu, Candan Demiroz Abakay, Betul Sevinir

**Affiliations:** 1 Department of Pediatric Hematology and Oncology, Izmir City Hospital, Şevket Ince Mahallesi, 2148/11, Sokak, No: 1/11, Bayraklı, Izmir 35540, Turkey; 2 Department of Pediatric Oncology, Faculty of Medicine, Uludag University, Gorukle Kampusu, Nilufer, Bursa 16059, Turkey; aytultemuroglu@uludag.edu.tr (A.T.);; 3 Department of Radiation Oncology, Faculty of Medicine, Uludag University, Gorukle Kampusu, Nilufer, Bursa 16059, Turkey

**Keywords:** pediatric nasopharyngeal carcinoma, TNM stage, late effects, survivorship, otolaryngologic toxicity

## Abstract

**Highlights:**

**What are the main findings?**

- Among this 20-year single institution cohort, pediatric nasopharyngeal cancer type III, according to the WHO classification, was an uncommon condition, which comprised 1.3% of all pediatric cancers, and patients generally had advanced stages at presentation.
- Overall survival rates were estimated at 72.7% for both 5 and 10 years, while poor prognosis was associated with AJCC stage IV disease and distant metastases at diagnosis.

**What are the implications of the main findings?**

- These studies point out the importance of risk assessment during the first stages of treatment, especially in the presence of stage IV pediatric nasopharyngeal carcinoma.
- The observed frequency of dysphagia, malnutrition, xerostomia, fibrosis, endocrine dysfunction, and otologic late effects underscores the importance of multidisciplinary long-term survivorship care.

**Abstract:**

**Objectives:** Pediatric nasopharyngeal carcinoma (NPC) is rare and often presents at an advanced stage. Although multimodal treatment can achieve favorable survival, long-term survivors may experience substantial treatment-related morbidity. We aimed to evaluate survival outcomes according to stage and metastatic status and to characterize late toxicity in a 20-year single-center pediatric NPC series. **Methods**. We retrospectively reviewed 24 pediatric patients diagnosed with NPC between 2003 and 2023. Histology was classified according to WHO criteria, and tumors were staged using the AJCC TNM system. Overall survival (OS) and event-free survival (EFS) were estimated using the Kaplan–Meier method. Survival distributions were compared using the log-rank test. Late treatment-related toxicities documented during follow-up were recorded descriptively. **Results:** Twenty-four patients with WHO type III NPC were included. Fourteen patients had stage III disease and 10 had stage IV disease; three had distant metastasis at diagnosis. The median follow-up duration was 50.5 months. At last follow-up, 19 patients were alive and five had died. The estimated 5- and 10-year OS rates were both 72.7%, and the corresponding EFS rates were both 63.7%. Stage IV disease and metastatic presentation were associated with inferior OS. Dysphagia, malnutrition, xerostomia, fibrosis, hypothyroidism, and deafness were the most frequently recorded adverse health effects. **Conclusions:** This 20-year single-center experience shows that AJCC stage and metastatic status remain key determinants of survival in pediatric NPC. The high burden of late treatment-related complications highlights the importance of integrating long-term multidisciplinary survivorship surveillance into the care of pediatric NPC survivors.

## 1. Introduction

Nasopharyngeal carcinoma (NPC) is an uncommon childhood malignancy accounting for only 1% of all cancers among children [1,2]. While NPCs among adults may present any time from childhood through to adulthood, in children, NPCs tend to occur at puberty, show significant EBV correlation, and WHO type III undifferentiated non-keratinizing carcinoma predominates [2,3,4]. It usually presents itself with non-specific complaints such as enlarged cervical nodes, nasal blockage, otitis, and headaches, among others, with many patients presenting with locally advanced or metastatic illness upon diagnosis [1,4,5].

Because surgical resection is rarely feasible, combined radiotherapy and platinum-based chemotherapy form the cornerstone of treatment. Long-term overall survival in pediatric NPC now exceeds 70–80% in many series [2,6,7,8]. As an increasing number of children survive into adulthood, attention has shifted toward late treatment-related morbidity. Radiotherapy to the developing head and neck, combined with platinum-based chemotherapy, is associated with xerostomia, dysphagia, nutritional problems, endocrine dysfunction, hearing impairment, fibrosis, and neurologic sequelae that may persist for years and impair quality of life [1,2,8,9].

Most pediatric NPC reports focus primarily on survival outcomes, treatment response, or selected individual late effects. However, single-institution data describing both long-term survival patterns and the spectrum of late treatment-related toxicities remain limited, particularly in real-world cohorts treated over extended time periods. We therefore reviewed 20 years of single-center experience with pediatric WHO type III NPC, reporting survival according to AJCC stage and metastatic status together with a descriptive profile of late treatment-related toxicities. By integrating survival outcomes with late morbidity data, this study aims to contribute real-world evidence to the limited literature on long-term outcomes and survivorship burden in pediatric NPC.

## 2. Materials and Methods

This retrospective single-center study included pediatric patients diagnosed with nasopharyngeal carcinoma and treated at the Department of Pediatric Oncology, Uludag University Faculty of Medicine, between January 2003 and December 2023. Medical records were reviewed to identify eligible patients. Patients were eligible if they were aged 18 years or younger at diagnosis and had sufficient clinical, treatment, and outcome data. Patients with inadequate treatment or outcome information were excluded. The experiment conformed to the guidelines of the Declaration of Helsinki and was supported by the Uludag University Clinical Research Ethics Committee (approval no: 2026/123/5-3; date: 4 March 2026).

Demographic data consisting of age at diagnosis and gender were extracted from clinical records. Clinical parameters on presentation, including cervical lymphadenopathy, nasal blockage, recurring ear infections, headache, neck enlargement, and lack of a specific symptom related to the primary diagnosis, were noted. Histopathological subtype, TNM stage, chemotherapy regimen, radiotherapy technique, relapse status, salvage treatment, follow-up duration, and survival status were also collected. Follow-up was calculated from the date of diagnosis to the date of last contact or death.

Histology was classified according to WHO criteria, and tumors were staged using the AJCC TNM staging system. During the study period, 28 pediatric patients with nasopharyngeal malignancy were identified. One patient with nasopharyngeal papillary adenocarcinoma treated with surgery alone was excluded because of its distinct histology, biological behavior, and treatment strategy. Three additional patients were excluded because of insufficient clinical, treatment, or follow-up data. Therefore, the final analytic cohort consisted of 24 patients with WHO type III undifferentiated non-keratinizing nasopharyngeal carcinoma, all of whom received multimodal treatment including chemotherapy and radiotherapy.

First-line treatment consisted of multimodal therapy, including induction chemotherapy followed by radiotherapy. Induction chemotherapy was administered using an institutional cisplatin-based regimen consisting of bleomycin, epirubicin, and cisplatin. This regimen reflected the institutional treatment standard for childhood nasopharyngeal carcinoma adopted at the start of the study period, when bleomycin-, anthracycline-, and platinum-containing induction schemes were in common use for this disease; it was retained for consistency across the cohort, predating the broader adoption of cisplatin plus 5-fluorouracil-based induction in contemporary pediatric protocols. Nineteen patients received intensity-modulated radiotherapy and five received conventional radiotherapy. Patients who developed relapse received salvage chemotherapy with the ICE regimen, including ifosfamide, etoposide, and carboplatin. Primary treatment and salvage treatment were reported separately.

Treatment response was evaluated during and/or after treatment using clinical and radiological assessments and was classified according to WHO response criteria. Complete response was defined as the disappearance of all evident disease. Partial response was defined as a reduction of more than 50% in measurable tumor burden. Stable disease or no response was defined as a reduction of less than 50% without evidence of progression. Progressive disease was defined as an increase in tumor burden or the appearance of new lesions. Recurrence or relapse was defined as the reappearance of disease after achieving complete or partial response at the end of planned therapy.

Overall survival was defined as the time from diagnosis to death from any cause or last follow-up. Event-free survival was defined as the time from diagnosis to the first occurrence of relapse, progression, or death from any cause. Patients without an event were censored at the date of last follow-up.

Late treatment-related toxicities were defined as chronic complications persisting or arising after completion of therapy and documented during follow-up. The following late effects were recorded as present or absent: xerostomia, dysphagia, malnutrition, hypothyroidism, hearing loss, fibrosis, neuropathy, recurrent otitis, and hypernasal speech. Late effects were ascertained from the documentation of the treating multidisciplinary team during routine survivorship follow-up. Dysphagia and hypernasal speech were based on clinical evaluation, including documentation by speech and swallowing assessment when available, rather than on a uniform instrumental swallowing study. Malnutrition was recorded when documented by the treating team on the basis of weight loss, impaired oral intake, or the need for nutritional support. Hypothyroidism was defined by abnormal thyroid function tests and/or the documented initiation of thyroid hormone replacement. Hearing loss was recorded when documented clinically, and was confirmed by audiometry and the need for hearing aids where available. Fibrosis and recurrent otitis were recorded on the basis of clinical examination, and neuropathy on the basis of documented clinical findings. Because of the retrospective design and the absence of uniform severity grading across the study period, late effects were reported descriptively as frequencies and percentages.

### Statistical Analysis

Descriptive statistics were used to summarize demographic, clinical, treatment-related, survival, and toxicity data. Continuous variables were expressed as median and range or mean ± standard deviation, as appropriate. Categorical variables were expressed as frequencies and percentages. Overall survival and event-free survival were estimated using the Kaplan–Meier method, and survival distributions were compared across AJCC stage, nodal stage, metastatic status, T stage, sex, age group, and radiotherapy technique using the log-rank test. Since the sample size was small, the toxicity results were described but not statistically compared using a statistical model. Two-sided *p*-values < 0.05 were considered statistically significant. The analysis was done using IBM SPSS Statistics, Version 22.0.

## 3. Results

A cohort of 24 children who were diagnosed with nasopharyngeal carcinoma was selected for the research. Within the same period, 1814 children who were diagnosed with either solid tumors or hematologic malignancies were admitted to our hospital, suggesting that the proportion of nasopharyngeal carcinoma among childhood cancers was 1.3%. The cohort comprised 14 males and 10 females, with a median age at diagnosis of 14 years (range, 10–18 years). The most common presenting findings were cervical lymphadenopathy in 10 patients (41.7%), neck swelling in 4 (16.7%), recurrent otitis in 3 (12.5%), and headache in 3 (12.5%). Six patients had no dominant disease-specific presenting symptom documented in the medical records. The median duration of symptoms before hospital admission was 3.5 months (range, 1–7 months).

All patients had WHO type III undifferentiated non-keratinizing carcinoma. Most patients had regional and/or locally advanced cancer. At diagnosis, one patient had bone metastasis and two patients had lung metastases. According to TNM staging, 17 patients had T2 disease and 7 had T3 disease; 14 had N2 disease and 10 had N3 disease; and 3 patients had M1 disease. According to AJCC stage, 14 patients had stage III disease and 10 had stage IV disease (Table 1).

All patients received induction chemotherapy with this cisplatin-based regimen, with a median of 3 cycles (range, 2–6 cycles). Sixteen patients received adjuvant chemotherapy after radiotherapy, with 2–3 additional cycles. Radiotherapy was planned to encompass the primary nasopharyngeal tumor together with bilateral cervical and supraclavicular nodal regions. The prescribed dose of 50–60 Gy (mean, 54.3 Gy) was delivered to the primary tumor and the involved cervical lymph nodes, whereas the uninvolved cervical and supraclavicular regions received prophylactic irradiation of 40–50 Gy. For patients treated with intensity-modulated radiotherapy, target volumes were delineated on planning computed tomography, and dose constraints to organs at risk (including the spinal cord, brainstem, parotid glands, cochleae, and optic apparatus) were applied according to the institutional protocol in effect at the time of treatment; for patients treated with conventional radiotherapy in the earlier study period, organ-at-risk sparing was more limited because formal dose–volume constraints were not routinely available. Mean radiotherapy duration was 7 weeks (6–12 weeks range), while the median number of fractions was 30 (22–33 range) (Table 2).

Nodal stage was not significantly associated with overall survival (Table 3). Sex, age group, T stage, and radiotherapy technique were also not significantly associated with overall survival. Given the small sample size and the limited number of events, all subgroup survival comparisons should be regarded as exploratory, and non-significant comparisons cannot be interpreted as evidence of no difference.

At the end of planned therapy, complete response was documented in 19 patients (79.2%), partial response in 4 patients (16.7%), and progressive disease in 1 patient (4.2%), who had distant metastatic disease. Relapse occurred in five patients: two had local or intracranial relapse, and three developed distant metastatic relapse involving bone alone, bone and liver, or bone and lung. Among the five patients who died, three died after relapse because of lack of response to subsequent salvage treatment. Two patients died with primary refractory/progressive disease; in one of them, infection contributed to death in the setting of treatment-related toxicity and advanced disease.

**Figure 1:**
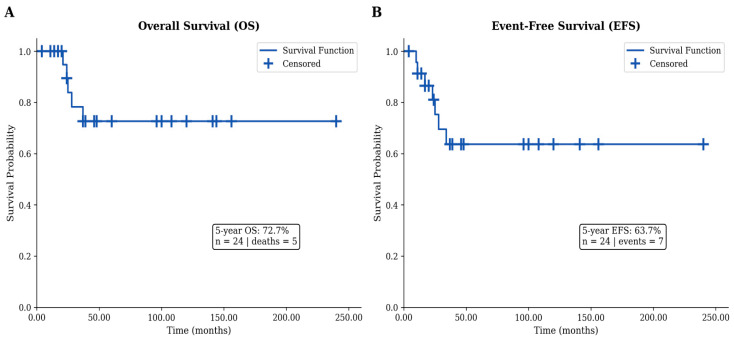
Kaplan–Meier survival curves for the entire cohort. Kaplan–Meier curves showing overall survival (OS) and event-free survival (EFS) in 24 pediatric patients with WHO type III nasopharyngeal carcinoma. The estimated 5- and 10-year OS rates were both 72.7%, and the estimated 5- and 10-year EFS rates were both 63.7%. No deaths were observed after 37 months of follow-up, and no additional EFS events were observed after 34 months. OS, overall survival; EFS, event-free survival. Censored observations, indicated by plus signs, represent patients who were alive or event-free at the last follow-up.

**Figure 2:**
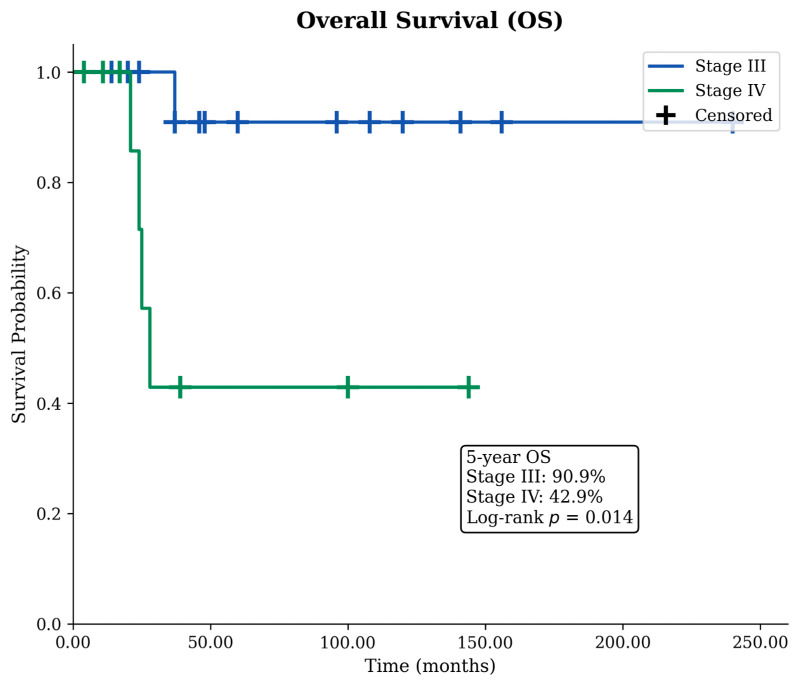
Overall survival according to AJCC stage. Kaplan–Meier curves comparing overall survival between patients with AJCC stage III and stage IV disease. The estimated 5-year OS rate was 90.9% for stage III disease and 42.9% for stage IV disease. Survival differed significantly between the two groups using the log-rank test (*p* = 0.014). AJCC, American Joint Committee on Cancer; OS, overall survival. Censored observations, indicated by plus signs, represent patients who were alive at the last follow-up.

The estimated 5- and 10-year event-free survival rates were both 63.7%, based on seven events (Figure 1). No additional event-free survival events were observed after 34 months of follow-up. Late treatment-related toxicities were frequently documented during follow-up. Survival also differed significantly according to AJCC stage, with an estimated 5-year OS of 90.9% for stage III disease compared with 42.9% for stage IV disease (log-rank p = 0.014) (Figure 2). The most common late effects were dysphagia in 13 patients (54.2%), malnutrition in 13 (54.2%), xerostomia in 11 (45.8%), fibrosis in 10 (41.7%), and hypothyroidism in 8 (33.3%). Hearing loss occurred in 7 patients (29.2%), of whom 6 required hearing aids. Hypernasal speech was observed in 6 patients (25.0%), recurrent otitis in 5 (20.8%), and neuropathy in 5 (20.8%) (Table 4). Regarding other late effects specifically assessed, no cardiac toxicity (e.g., reduction in left ventricular ejection fraction or clinical cardiac dysfunction) was documented in any patient during follow-up.

## 4. Discussion

In this single-center case series of pediatric NPC spanning two decades, the estimated 5- and 10-year OS of 72.7% was within the broad range reported in pediatric NPC series and comparable with several historical and real-world cohorts treated with combined chemotherapy and radiotherapy. Previous studies have reported widely variable survival outcomes, with OS rates ranging from approximately 55% to 90% and DFS/EFS rates from approximately 60% to 77%, depending on disease stage, treatment period, chemotherapy regimen, radiotherapy technique, and the inclusion of metastatic patients [6,8,10,11,12,13,14]. Although contemporary cooperative-group protocols incorporating induction chemotherapy, radiotherapy, and risk-adapted treatment approaches have reported 5-year OS rates exceeding 90% in selected pediatric and adolescent cohorts, our results should be interpreted in the context of a real-world single-center cohort, inclusion of metastatic patients, treatment evolution over a 20-year period, and the small sample size inherent to this rare disease [7,8]. In our cohort, stage IV disease and metastatic presentation were the main factors associated with poorer survival, whereas sex, age group, T stage, nodal stage, and radiotherapy technique were not significantly associated with survival.

The adverse impact of metastatic disease observed in our series is consistent with previous pediatric NPC studies and population-based analyses showing that disease extent remains a major determinant of outcome [4,5,10,14,15,16]. In the present cohort, all three patients with M1 disease died, whereas survival was substantially better among non-metastatic patients. This finding highlights that, despite improvements in imaging, radiotherapy techniques, and multimodal treatment, distant disease remains a major therapeutic challenge in pediatric NPC. The relatively narrow difference between EFS and OS also suggests that most treatment failures occurred early, with poor outcomes after relapse in several patients. These findings support considering metastatic or relapsed pediatric NPC as a clinically high-risk group in future multicenter studies and treatment strategies [1,2,10]. Induction chemotherapy regimens for pediatric NPC have varied across previous single-institution series and historical treatment periods [7,8,11,15,16]. In the present cohort, treatment reflected the institutional cisplatin-based induction approach used during the study period.

Although nodal stage was not statistically associated with overall survival in our cohort, the lower 5-year OS observed in patients with N3 disease compared with those with N2 disease suggests a clinically relevant adverse trend (N3%50 vs. N2%84). This difference should be interpreted cautiously because of the small sample size and limited number of events, but it supports further evaluation of the prognostic role of nodal burden in larger pediatric NPC series [10,15,16].

Against this survival background, late treatment-related toxicities were frequent. More than half of the patients experienced dysphagia and malnutrition, and nearly half had xerostomia and fibrosis. Such outcomes coincide with the established late toxicities from combined head and neck radiotherapy and platinum chemotherapy, such as salivary gland hypofunction, dysphagia, soft tissue fibrosis, endocrine abnormalities, and otologic toxicities [1,2,8,11,15]. From previous studies involving pediatric NPC patients, it is known that hypothyroidism, xerostomia, ototoxicity, and fibrosis are some of the most common late complications, which may increase with more follow-up time [8,11,12,15,17]. Among our group, otolaryngologic problems and malnutrition were especially common, thus indicating the point that survival does not reflect all of the morbidity caused by the treatment of pediatric NPC.

The relatively high rate of late toxicity must be considered against the background of the development of the therapy during the twenty-year observation period. No significant difference in overall survival was observed between the IMRT and conventional radiotherapy groups; however, this finding should not be interpreted as evidence of equivalence between the two techniques. With only five patients treated with conventional radiotherapy, this comparison is markedly underpowered, and the absence of a statistically significant difference may reflect insufficient statistical power and a possible type II error rather than true equivalence. Beyond survival, IMRT is generally regarded as an advance aimed at reducing treatment-related morbidity while maintaining comparable disease control [17,18,19,20,21]. Therefore, the presence of participants having undergone conventional radiation therapy before IMRT can explain the observed cases of xerostomia, fibrosis, dysphagia, and other late effects [17].

The group showed an important incidence of otologic toxicity. Hearing impairment occurred in about one third of the patients, most of whom had to use amplification equipment. This is consistent with the known ototoxic effects seen with cisplatin therapy and irradiation of the nasopharynx [1,2,17,22]. For otolaryngology, this study supports audiologic screening for these patients to facilitate early rehabilitation and monitoring of their speech and swallowing function [22,23]. Likewise, the high prevalence of swallowing problems and malnutrition suggests that the nutritional and swallowing status of these survivors needs to be continuously monitored instead of waiting for a clinically obvious decline in condition [17,23].

While no information on severity classification could be gathered due to the retrospective nature of the study, the incidence and characteristics of late effects show their clinical significance for survivorship problems. Therefore, it is crucial to perform multidisciplinary monitoring of patients regarding dysphagia, nutrition, endocrine, and auditory late effects [1,2,22,23].

In terms of looking into the future, the high survivorship burden in this group highlights the importance of risk adaptation and response adaptation in children suffering from nasopharyngeal carcinoma (NPC). Newer approaches have adopted EBV-DNA measurement as a method of assessing the risks and responses of the patients, whereas reduction in the radiotherapy dose, if used appropriately in good responders, is also being considered [1,2,18,19,20,21]. Contemporary pediatric NPC strategies increasingly integrate plasma EBV DNA monitoring at diagnosis and during treatment with response-adapted approaches, in which the intensity of consolidation and the radiotherapy dose are tailored to the response to induction chemotherapy, allowing radiotherapy dose de-escalation in selected good responders in order to reduce late toxicity without compromising disease control [18,19,20,21]. In our cohort, EBV-related diagnostic and prognostic markers were not uniformly available across the study period, so response-adapted and EBV-guided strategies could not be applied or evaluated; this should be regarded as a limitation of the present series and as a clear priority for future prospective and multicenter studies. As pediatric NPC is quite uncommon, further multicenter research must focus on toxicity grading using CTCAE, risk stratification using EBV DNA testing, and multidisciplinary survivorship surveillance [1,2,20,21,22,23].

There are numerous limitations in this study. For instance, this is a retrospective, single-center case series with few cases owing to the rarity of pediatric NPC. Therefore, the survival comparisons should be interpreted cautiously, and the limited number of events precluded robust multivariable prognostic modeling. Second, treatment protocols and radiotherapy techniques evolved over the 20-year study period, introducing residual heterogeneity and limiting causal conclusions regarding treatment-specific outcomes or toxicity. To reduce biological heterogeneity, the analysis was restricted to patients with WHO type III undifferentiated non-keratinizing nasopharyngeal carcinoma, and primary and salvage treatment settings were reported separately. Third, late effects were ascertained from clinical documentation, and consistent CTCAE-based severity grading was not available across the entire study period. Therefore, late toxicities were reported descriptively as documented clinical events, with functional indicators such as hearing-aid requirement used when available. Because follow-up duration varied and some patients died early, the reported late toxicity frequencies may underestimate the true burden among long-term survivors. EBV-related diagnostic or prognostic markers were not uniformly available during the study period; therefore, EBV status could not be incorporated into the outcome analyses. Trismus was not systematically recorded in our retrospective dataset and therefore could not be reliably reported; this represents a limitation of the present study. In addition, because the institutional bleomycin-, epirubicin-, and cisplatin-based induction regimen differs from contemporary cisplatin plus 5-fluorouracil-based protocols, direct comparisons with modern treatment cohorts should be made cautiously.

## 5. Conclusions

In this 20-year single-center case series of pediatric WHO type III nasopharyngeal carcinoma, long-term survival differed significantly according to AJCC stage and metastatic status. Stage III disease was associated with favorable long-term outcomes, whereas stage IV disease and metastatic presentation remained associated with poorer survival. Late complications arising from the treatment were prevalent, specifically including swallowing problems, malnutrition, dry mouth, tissue scarring, hypothyroidism, and deafness. Such results highlight the importance of having structured survivorship care that involves a pediatric otolaryngology consultation, nutritional evaluation, endocrine monitoring, and audiological screening.

## Figures and Tables

**Table 1:** Baseline clinical and staging characteristics of the cohort (*n* = 24).

Variable	Value
Total cohort	24
Male sex	14 (58.3)
Female sex	10 (41.7)
Age at diagnosis, years, median (range)	14 (10–18)
Symptom duration before admission, months, median (range)	3.5 (1–7)
Cervical lymphadenopathy	10 (41.7)
Neck swelling	4 (16.7)
Recurrent otitis	3 (12.5)
Headache	3 (12.5)
No dominant disease-specific presenting symptom documented	6 (25.0)
WHO type III undifferentiated non-keratinizing carcinoma	24 (100)
T2 disease	17 (70.8)
T3 disease	7 (29.2)
N2 disease	14 (58.3)
N3 disease	10 (41.7)
M0 disease	21 (87.5)
M1 disease	3 (12.5)
Bone metastasis at diagnosis	1 (4.2)
Lung metastasis at diagnosis	2 (8.3)
AJCC stage III	14 (58.3)
AJCC stage IV	10 (41.7)

Data are presented as *n* (%) unless otherwise indicated. Percentages were calculated using the total cohort as the denominator (*n* = 24). Presenting findings were based on medical-record documentation. AJCC, American Joint Committee on Cancer; WHO, World Health Organization.

**Table 2:** Treatment characteristics by treatment setting.

Category	Treatment Characteristic	Value
Primary treatment	Combined chemotherapy and radiotherapy	24 (100)
First-line chemotherapy	Cisplatin- based regimen	24 (100)
Induction chemotherapy cycles, median (range)		3 (2–6)
Adjuvant chemotherapy after radiotherapy		16 (66.7)
Adjuvant chemotherapy cycles		2–3
Radiotherapy technique	IMRT: 19 (79.2) Conventional radiotherapy: 5 (20.8)	
Radiation dose to primary tumor and involved cervical lymph nodes		50–60 Gy
Median radiation dose		54.3 Gy
Prophylactic irradiation to uninvolved cervical and supraclavicular regions		40–50 Gy
Radiotherapy duration, weeks, mean (range)		7 (6–12)
Radiotherapy fractions, median (range)		30 (22–33)
Relapse/salvage setting	Relapsed patients	5 (20.8)
Salvage chemotherapy among relapsed patients	ICE regimen	5/5 (100)

Data are presented as *n* (%) unless otherwise indicated. Percentages were calculated using the total cohort as the denominator (*n* = 24), except for salvage chemotherapy, which was calculated among relapsed patients. ICE, ifosfamide–etoposide–carboplatin; IMRT, intensity-modulated radiotherapy. The institutional cisplatin-based regimen consisted of bleomycin, epirubicin, and cisplatin.

**Table 3:** Survival by stage, metastatic status, and nodal stage.

Group	*n*	Deaths	5-Year OS	Log-Rank *p*
Stage III	14	1	90.9%	0.014
Stage IV	10	4	42.9%	
M0	21	2	86.7%	<0.001
M1	3	3	0%	
N2	14	2	83.9%	0.123
N3	10	3	50.0%	

Overall survival was estimated using the Kaplan–Meier method, and survival distributions were compared using the log-rank test. Percentages represent estimated 5-year overall survival rates. American Joint Committee on Cancer; OS, overall survival. Given the small sample size, all survival subgroup comparisons were considered exploratory.

**Table 4:** Late treatment-related toxicities.

Toxicity	*n* (%)
Dysphagia	13 (54.2)
Malnutrition	13 (54.2)
Xerostomia	11 (45.8)
Fibrosis	10 (41.7)
Hypothyroidism	8 (33.3)
Hearing loss (hearing aid 6/7)	7 (29.2)
Hypernasal speech	6 (25.0)
Recurrent otitis	5 (20.8)
Neuropathy	5 (20.8)

Data are presented as *n* (%). Percentages were calculated using the total cohort as the denominator (*n* = 24). Late toxicities were recorded as present or absent based on clinical documentation during follow-up.

## Data Availability

The datasets generated during and/or analyzed during the current study are available from the corresponding author on reasonable request.
